# Non-alcoholic fatty liver disease among patients with sleep disorders: a Nationwide study of Taiwan

**DOI:** 10.1186/s12876-020-1178-7

**Published:** 2020-02-10

**Authors:** Yu-Ting Wei, Peng-Yi Lee, Cheng-Yu Lin, Hsuan-Ju Chen, Che-Chen Lin, Jin-Shang Wu, Yin-Fan Chang, Chen-Long Wu, How-Ran Guo

**Affiliations:** 1 Department of Environmental and Occupational Health, College of Medicine, National Cheng Kung University, No.138, Sheng Li Road, Tainan, 70403 Taiwan, Republic of China; 2 Department of Occupational and Environmental Medicine, National Cheng Kung University Hospital, College of Medicine, National Cheng Kung University, No.138, Sheng Li Road, Tainan, 70403 Taiwan, Republic of China; 3 Department of Family Medicine, National Cheng Kung University Hospital, College of Medicine, National Cheng Kung University, No.138, Sheng Li Road, Tainan, 70403 Taiwan, Republic of China; 4 Occupational Safety, Health, and Medicine Research Center, National Cheng Kung University, No.138, Sheng Li Road, Tainan, 70403 Taiwan, Republic of China; 5 Preventive Medicine Center, Taichung Tzu Chi Hospital, Buddhist Tzu Chi Medical Foundation, No. 88, Sec. 1, Fengxing Road, Taichung, 42743 Taiwan, Republic of China; 6 Department of Radiation Oncology, China Medical University Hospital, No. 2, Yude Road, Taichung, 40447 Taiwan, Republic of China; 7 Department of Radiation Oncology, China Medical University Beigang Hospital, No.123, Sinde Road, Yunlin, 65152 Taiwan, Republic of China; 8 Department of Otolaryngology, National Cheng Kung University Hospital, College of Medicine, National Cheng Kung University, No.138, Sheng Li Road, Tainan, 70403 Taiwan, Republic of China; 9 Sleep Medicine Center, National Cheng Kung University Hospital, College of Medicine, National Cheng Kung University, No.138, Sheng Li Road, Tainan, 70403 Taiwan, Republic of China; 10 Management Office for Health Data, China Medical University Hospital, No.2, Yude Road, North District, Taichung, 40447 Taiwan, Republic of China; 11 College of Medicine, China Medical University, No.91, Hsueh-Shih Road, Taichung, 40402 Taiwan, Republic of China; 12 Healthcare Service Research Center, Taichung Veterans General Hospital, No.1650 Taiwan Boulevard Sect. 4, Taichung, 40705 Taiwan, Republic of China; 13 Department of Family Medicine, College of Medicine, National Cheng Kung University, No. 1 University Road, Tainan, 70101 Taiwan, Republic of China; 14 Department of Occupational and Environmental Medicine, College of Medicine, National Cheng Kung University, No.138, Sheng Li Road, Tainan, 70403 Taiwan, Republic of China

**Keywords:** Epidemiology, Sleep disorders, Nonalcoholic fatty liver disease, Population-based cohort study

## Abstract

**Background:**

Nonalcoholic fatty liver disease (NAFLD) is one of the most common chronic liver diseases. Studies have shown that sleep apnea is associated with NAFLD. However, studies on the association between sleep disorders in general and NAFLD are limited. We conducted a nationwide population-based longitudinal study to evaluate this potential association.

**Methods:**

We identified patients diagnosed with sleep disorders in the years 2000 through 2005 in Taiwan using the National Health Insurance Research Database and selected an equal number of patients without sleep disorders from the same database as the comparison cohort. The patients were followed from the index date to the diagnosis of NAFLD or the end of 2013. We used Cox proportional hazards models to estimate the risk of NAFLD associated with sleep disorders.

**Results:**

A total of 33,045 patients with sleep disorders were identified. The incidence of NAFLD was 14.0 per 10,000 person-year in patients with sleep disorders and 6.2 per 10,000 person-year in the comparison cohort. The adjusted hazard ratio (AHR) of NAFLD associated with sleep disorders was 1.78 (95% confidence interval [95%CI]: 1.46–2.16), and other independent risk factors included male sex (AHR = 1.31, 95%CI: 1.12–1.54), age 40–59 years (AHR = 1.49, 95%CI: 1.21–1.82), and dyslipidemia (AHR = 2.51, 95%CI: 2.08–3.04). In the subgroup analyses, both patients with (AHR = 2.24, 95%CI: 1.05–4.77) and without (AHR = 1.77, 95%CI: 1.46–2.15) sleep apnea had an increased risk of NAFLD.

**Conclusions:**

Sleep disorders are associated with NAFLD, even in patients without sleep apnea. Further studies are warranted to explore the mechanisms of the association.

## Background

Nonalcoholic fatty liver disease (NAFLD) is a rapidly growing public health threat globally. NAFLD is believed to be a hepatic border of metabolic syndrome and is associated with many metabolic changes such as insulin resistance. The proposed mechanisms of NAFLD include predisposition and intake of higher energy, which result in liver damages ranging from steatosis to nonalcoholic steatohepatitis, advanced fibrosis, and eventually, cirrhosis [1, 2]. The prevalence of NAFLD in the general population is 20 to 30% in the Western world [3], and 5 to 40% across the Asia-Pacific region [4–6]. NAFLD is also an emerging liver disease in Taiwan, with prevalence ranging from 11.4 to 41% [7].

Sleep apnea is a kind of sleep disorders (SD). It refers to momentary, often cyclical, cessations in breathing rhythm, sufficient to cause significant arterial hypoxemia and hypercapnia [8]. Many studies have been conducted on the association between sleep apnea and NAFLD, and the pooled odds ratios were approximately 2 to 3 in meta-analyses [9, 10]. However, sleep apnea constitutes only a portion of sleep disturbance. SD, including short sleep duration, poor sleep quality, etc. [11, 12], are common in the general population [13, 14]. For example, more than 25% of the Taiwanese adults suffer from insomnia [15]. Patients with SD are at increased risks of obesity, insulin resistance, dyslipidemia, hypertension, diabetes mellitus [16], and cardiovascular disease [17], which have all been reported to be associated with NAFLD [18, 19].

Studies on the risk of NAFLD associated with SD are limited. Using “sleep disorder” and “non-alcoholic fatty liver disease” as the keywords to search the literature indexed in the PubMed, we found that most of the previous studies were focused on sleep apnea, and all the limited studies on NAFLD associated with SD in general were cross-sectional studies, which may fall prey to the problem of “inverse causation,” i.e. NAFLD being a cause instead of an outcome of SD. Therefore, we conducted a longitudinal study to evaluate the association between SD, including sleep apnea, and NAFLD.

## Methods

We conducted a retrospective population-based cohort study in Taiwan using the National Health Insurance system established by the Taiwanese government in 1995, which covers nearly all Taiwanese citizens. The National Health Insurance Research Database 2000 (NHIRD 2000) contains medical claim data from one million beneficiaries who were randomly selected in 2000. The cohort members have been followed up since the construction of the database, which included the registry of beneficiaries, disease registry profile, drug prescriptions, and other medical services. The disease registry profile recorded the disease history for each insured individual according to the International Classification of Diseases, Ninth Revision, Clinical Modification (ICD-9-CM). The database underwent de-identification before it was released for research use.

The target cohort are those who had SD (defined by having ICD-9-CM 307.4 or 780.5 among the diagnoses on the claims) for at least three consecutive months from 2000 to 2005. The index date of an SD patient was defined as the first diagnosis date. The same number of beneficiaries as those in the SD cohort were randomly selected from those who did not have SD from the same database as the comparison cohort. We excluded candidates who were under 20 years of age and who had a history of NAFLD (ICD-9-CM 571.8), liver cirrhosis (ICD-9-CM 571.2, 571.5, or 571.6), hepatitis B (ICD-9-CM V02.61, 070.20, 070.22, 070.30, or 070.32), hepatitis C (ICD-9-CM V02.62, 070.41, 070.44, 070.51, or 070.54), organic sleep disorders (ICD-9-CM 327), or narcolepsy (ICD-9-CM 347) before the index date. Both cohorts were followed up from the index date to the date when NAFLD was diagnosed or the end of 2013.

To control potential confounding factors, we collected data on diabetes (ICD-9-CM 250), dyslipidemia (ICD-9-CM 272), hypertension (ICD-9-CM 401–405), ischemic heart disease (IHD; ICD-9-CM 410–414), depression (ICD-9-CM 296.2, 296.3, 300.4, or 311), and anxiety (ICD-9-CM 300).

We present continuous variables as mean ± standard deviation and categorical variables as number (percentage). Differences in categorical variables between groups were evaluated using Pearson’s chi-square tests, and Student’s t-tests were used to evaluate differences in continuous variables. The incidence rate (IR) of NAFLD was calculated as the number of events divided by the person-year observed. We further plotted the cumulative incidence curves for the SD and compared the two cohorts using the Kaplan-Meier method. The log-rank test was used to evaluate the difference.

We used Cox proportional hazards regression models to obtain the hazard ratios (HRs) associated with SD. Univariate analyses were followed by multivariate analyses adjusting for sex, age, and comorbidities, including diabetes mellitus, dyslipidemia, hypertension, ischemic heart disease (IHD), depression, and anxiety. Furthermore, we divided the SD cohort into two subgroups, the sleep apnea group (ICD-9-CM 780.51, 780.53, or 780.57) and the non-apnea group (ICD-9-CM 307.4, 780.50, 780.52, 780.54, 780.55, 780.56, or 780.59), and conducted analyses separately.

In all statistical tests, the significant level was set at 0.05 (two-tailed). All statistical analyses were performed using the SAS 9.4 software (SAS Institute, Cary, NC, USA) or the R software. Our study protocol was reviewed and approved by the Ethics Review Board of the China Medical University Hospital (CMUH104-REC2–115).

## Results

A total of 33,045 patients were included in the SD cohort, and therefore the comparison cohort also had 33,045 members. The proportion of men was 39.7% in the SD cohort and 57.6% in the comparison cohort (*p* < 0.001) (Table 1). The mean age of the SD cohort was 12.3 years older than that of the comparison cohort (53.6 vs. 41.3 years, *p* < 0.001). The prevalence rates of the comorbidities of diabetes mellitus, dyslipidemia, hypertension, IHD, depression, and anxiety were all significantly higher in the SD cohort than those in the comparison cohort (all *p* < 0.001).

**Table 1:** Baseline demographic factors and comorbidities of study participants according to sleep disorder status

	Comparison Cohort		Sleep Disorder Cohort		
	(*N* = 33,045)		(*N* = 33,045)		
Characteristics	N	%	N	%	*p*-value^a^
Sex					< 0.001
Women	14,002	42.4	19,937	60.3	
Men	19,043	57.6	13,108	39.7	
Age (year)					< 0.001
20–39	17,696	53.6	7582	22.9	
40–59	10,817	32.7	13,271	40.2	
≥ 60	4532	13.7	12,192	36.9	
Mean (standard deviation)	41.3	(15.6)	53.6	(16.5)	< 0.001^b^
Comorbidities					
Diabetes	1529	4.6	4816	14.6	< 0.001
Dyslipidemia	2451	7.4	8474	25.6	< 0.001
Hypertension	4231	12.8	14,476	43.8	< 0.001
Ischemic Heart Disease	1659	5.0	8176	24.7	< 0.001
Depression	218	0.7	4111	12.4	< 0.001
Anxiety disorder	1120	3.4	12,870	39.0	< 0.001

^a^Chi-square test, unless otherwise noted; ^b^Student’s t-test

**Fig. 1:**
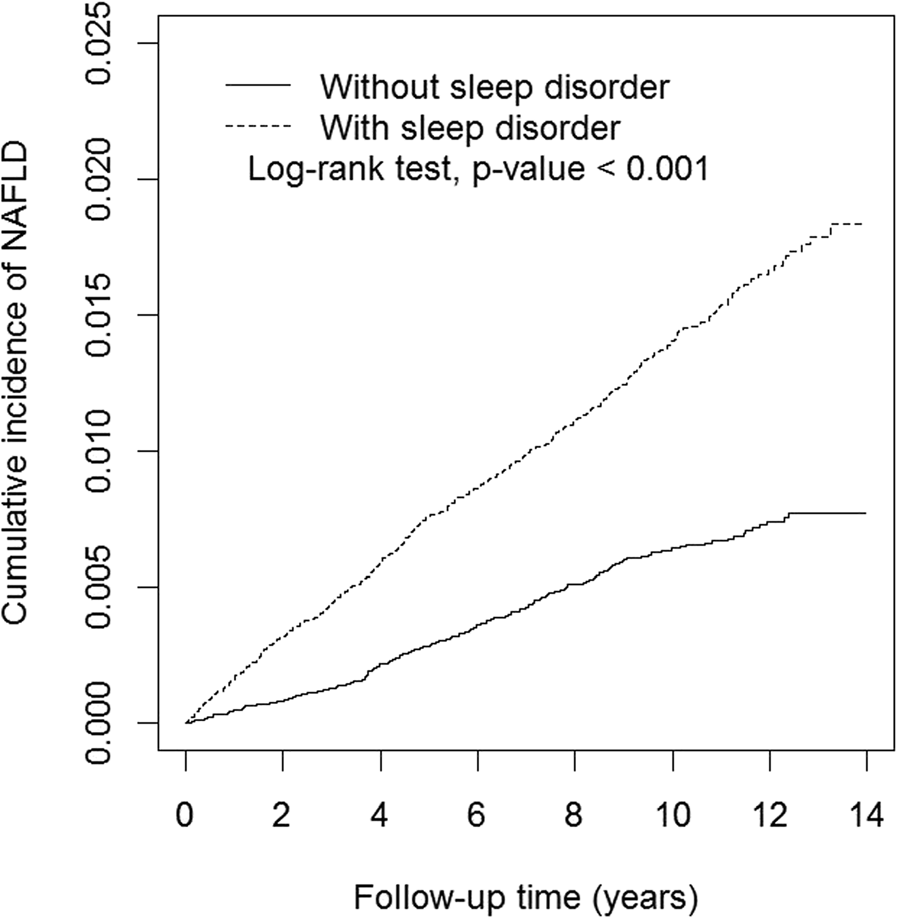
Cumulative incidence curves of non-alcoholic fatty liver disease for cohorts with and without sleep disorders

The IR of NAFLD was 14.0 per 10,000 person-year in the SD cohort and was only 6.2 per 10,000 person-year in the comparison cohort. The HR was 2.26 (95% confidence interval [95% CI]: 1.92–2.67) in the SD cohort with the comparison cohort as the reference (Table 2). After adjusting for age, sex, and comorbidities (diabetes, dyslipidemia, hypertension, IHD, depression, and anxiety), we found that patients with SD had an increased risk of developing NAFLD, with an adjusted HR (AHR) of 1.78 (95% CI: 1.46–2.15). In addition, there were significant higher risks associated with male sex (AHR = 1.31, 95% CI: 1.12–1.54), age 40–59 years (AHR = 1.49, 95% CI: 1.21–1.82), and dyslipidemia (AHR = 2.51, 95% CI: 2.08–3.04). However, the AHRs associated with diabetes (AHR = 1.04, 95% CI: 0.82–1.33) and hypertension (AHR = 1.02, 95% CI: 0.84–1.25) did not reach statistical significance in the multi-variate Cox proportional hazards regression analyses. The cumulative incidence of NAFLD in the SD cohort was significantly higher than that in the comparison cohort (*p* < 0.001) (Fig. 1).

**Table 2:** Hazard ratios of non-alcoholic fatty liver disease associated with sleep disorders and covariates

Characteristics	N	Event	Person-year	Incidence Rate (/10,000 person-year)	Hazard Ratio (95% Confidence Interval)			
					Univariate	*p*-value	Multivariate^a^	*p*-value
Sleep disorders								
No	33,045	206	333,121	6.2	Reference		Reference	
Yes	33,045	441	314,627	14.0	2.26 (1.92–2.67)	< 0.001	1.78 (1.46–2.16)	< 0.001
Sex								
Women	33,939	325	337,279	9.6	Reference		Reference	
Men	32,151	322	310,470	10.4	1.08 (0.92–1.26)	0.35	1.31 (1.12–1.54)	< 0.001
Age (year)								
20–39	25,278	166	260,647	6.4	Reference		Reference	
40–59	24,088	337	245,204	13.7	2.16 (1.79–2.60)	< 0.001	1.49 (1.21–1.82)	< 0.001
≥ 60	16,724	144	141,898	10.2	1.59 (1.27–1.99)	< 0.001	0.86 (0.66–1.13)	0.28
Comorbidities								
Diabetes								
No	59,745	556	592,827	9.4	Reference		Reference	
Yes	6345	91	54,922	16.6	1.77 (1.41–2.20)	< 0.001	1.04 (0.82–1.33)	0.73
Dyslipidemia								
No	55,165	411	544,066	7.6	Reference		Reference	
Yes	10,925	236	103,683	22.8	3.01 (2.56–3.53)	< 0.001	2.51 (2.08–3.04)	< 0.001
Hypertension								
No	47,383	407	479,123	8.5	Reference		Reference	
Yes	18,707	240	168,625	14.2	1.68 (1.43–1.97)	< 0.001	1.02 (0.84–1.25)	0.83
Ischemic Heart Disease								
No	56,255	529	560,892	9.4	Reference		Reference	
Yes	9835	118	86,856	13.6	1.44 (1.18–1.76)	< 0.001	0.86 (0.68–1.08)	0.19
Depression								
No	61,761	574	606,799	9.5	Reference		Reference	
Yes	4329	73	40,950	17.8	1.88 (1.47–2.40)	< 0.001	1.23 (0.94–1.62)	0.14
Anxiety								
No	52,100	437	514,026	8.5	Reference		Reference	
Yes	13,990	210	133,722	15.7	1.84 (1.56–2.17)	< 0.001	1.16 (0.95–1.42)	0.15

^a^Adjusted for sex, age (categorical), diabetes, dyslipidemia, hypertension, depression, anxiety, and ischemic heart disease in Cox proportional hazards regressions

In the stratified analyses (Table 3), the AHR in women was 1.82 (95% CI: 1.35–2.46), similar to that in men (AHR = 1.79, 95% CI: 1.38–2.32). At the age of 20–39, 40–59 and ≥ 60 years, the AHR was 1.71 (95% CI: 1.18–2.48), 1.89 (95% CI: 1.44–2.47) and 1.23 (95% CI: 0.79–1.91), respectively. All the AHRs in the groups without the comorbidity were significantly higher than those in the groups with the comorbidity. Specifically, the AHR was 1.84 (95% CI: 1.50–2.26) in patients without diabetes, 2.10 (95% CI: 1.67–2.65) in patients without dyslipidemia, 2.03 (95% CI: 1.61–2.57) in patients without hypertension, 1.88 (95% CI: 1.53–2.31) in patients without IHD, 1.81 (95% CI: 1.48–2.20) in patients without depression, and 1.82 (95% CI: 1.48–2.24) in patients without anxiety. None of the AHRs in the subgroups with those comorbidities reached statistical significance.

**Table 3:** Incidence rates and hazard ratios of non-alcoholic fatty liver disease according to sleep disorder status

	Comparison Cohort			Sleep Disorder Cohort			Compared to the Comparison Cohort			
							Hazard Ratio (95% Confidence Interval)			
Characteristics	Event	Person-year	IR	Event	Person-year	IR	Crude	*p*-value	Adjusted^a^	*p*-value
Sex										
Women	65	142,160	4.6	260	195,118	13.3	2.92 (2.22–3.83)	< 0.001	1.82 (1.35–2.46)	< 0.001
Men	141	190,961	7.4	181	119,509	15.2	2.04 (1.64–2.54)	< 0.001	1.79 (1.38–2.32)	< 0.001
Age (year)										
20–39	88	183,841	4.8	78	76,805	10.2	2.11 (1.55–2.86)	< 0.001	1.71 (1.18–2.48)	0.004
40–59	89	111,058	8.0	248	134,146	18.5	2.31 (1.81–2.94)	< 0.001	1.89 (1.44–2.47)	< 0.001
≥ 60	29	38,222	7.6	115	103,676	11.1	1.46 (0.97–2.20)	0.07	1.23 (0.79–1.91)	0.35
Comorbidities										
Diabetes										
No	186	320,194	5.8	370	272,633	13.6	2.34 (1.96–2.79)	< 0.001	1.84 (1.50–2.26)	< 0.001
Yes	20	12,927	15.5	71	41,995	16.9	1.11 (0.68–1.82)	0.68	1.17 (0.68–1.99)	0.57
Dyslipidemia										
No	153	309,909	4.9	258	234,156	11.0	2.24 (1.83–2.73)	< 0.001	2.10 (1.67–2.65)	< 0.001
Yes	53	23,212	22.8	183	80,471	22.7	0.99 (0.74–1.36)	0.99	1.03 (0.74–1.44)	0.85
Hypertension										
No	157	295,681	5.3	250	183,442	13.6	2.56 (2.10–3.13)	< 0.001	2.03 (1.61–2.57)	< 0.001
Yes	49	37,440	13.1	191	131,186	14.6	1.12 (0.82–1.53)	0.49	1.09 (0.78–1.52)	0.61
Ischemic heart disease										
No	190	319,112	6.0	339	241,780	14.0	2.35 (1.97–2.81)	< 0.001	1.88 (1.53–2.31)	< 0.001
Yes	16	14,009	11.4	102	72,847	14.0	1.23 (0.73–2.09)	0.44	1.04 (0.60–1.82)	0.88
Depression										
No	203	331,189	6.1	371	275,610.	13.5	2.19 (1.85–2.60)	< 0.001	1.81 (1.48–2.20)	< 0.001
Yes	3	1932	15.5	70	39,017	17.9	1.16 (0.37–3.68)	0.80	0.99 (0.31–3.17)	0.98
Anxiety										
No	191	322,595	5.9	246	191,432	12.9	2.17 (1.79–2.62)	< 0.001	1.82 (1.48–2.24)	< 0.001
Yes	15	10,526	14.3	195	123,196	15.8	1.11 (0.66–1.88)	0.69	1.04 (0.61–1.77)	0.88

*IR* Incidence rate per 10,000 person-year

^a^Model mutually adjusted for sex, age (categorical), diabetes, dyslipidemia, hypertension, depression, anxiety, and ischemic heart disease

Amongst the SD cohort, the sleep apnea group had an increased risk of NAFLD, with an AHR of 2.24 (95% CI: 1.05–4.77) after adjustment for age, sex, and the comorbidities (Table 4). Nonetheless, the non-apnea group also had an increased risk of NAFLD, with an AHR of 1.77 (95% CI: 1.46–2.15).

**Table 4:** Incidence rates and hazard ratios of non-alcoholic fatty liver disease in different subgroups

Subgroup	N	Event	Person-year	Incidence Rate (/10,000 person-year)	Hazard Ratio (95% Confidence Interval)			
					Crude	*p*-value	Adjusted^a^	*p*-value
Comparison Cohort	33,045	206	333,121	6.2	Reference		Reference	
Sleep Disorder Cohort								
Apnea Group	388	7	3751	18.7	3.01 (1.42–6.39)	0.004	2.24 (1.05–4.77)	0.04
Non-apnea Group	32,657	434	310,877	14.0	2.25 (1.91–2.66)	< 0.001	1.77 (1.46–2.15)	< 0.001

^a^Model adjusted for sex, age (categorical), diabetes, dyslipidemia, hypertension, depression, anxiety, and ischemic heart disease

## Discussion

This nationwide retrospective population-based cohort study found that patients with SD had a significantly higher risk of developing NAFLD. The increased risk of NAFLD was observed not only in the subgroups of SD patients with sleep apnea, but also in SD patients without sleep apnea. Previous studies on the association between SD and NAFLD were mostly on sleep apnea. Using “sleep disorder” and “non-alcoholic fatty liver disease” as the key words to search the literature indexed in the PubMed, we found five studies on the association between NAFLD and SD in general. The National Health and Nutrition Examination Survey (NHANES) in 2005 to 2010 in the U.S. found that SD was associated with a 1.4 times higher risk of NAFLD [20]. A study of 69,463 middle-aged Korean workers and their spouses found that short sleep duration and poor sleep quality were significantly associated with an increased risk of NAFLD [21]. A study of 46 patients with biopsy-proven NAFLD and 22 healthy controls also found that in the NAFLD patients, sleep duration was shortened, sleep onset was delayed, and sleep quality was poor [22]. A study of 2172 people in Japan found that the prevalence of NAFLD tended to decrease as sleep duration increased in men, but in women, and that it was lowest in the group with a sleep duration of 6 to ≤7 h and highest in the groups with sleep durations of ≤6 and > 8 h [23]. In younger populations, a study of 708 non-diabetic youngsters found that sleep shortage was associated with the presence of NAFLD [24]. In general, findings in those studies are compatible with our finding of an association between SD and NAFLD.

We observed an AHR of 2.24 for developing NAFLD in the SD patients with sleep apnea, which is consistent with the pooled odds ratios (between 2 and 3) obtained in meta-analyses [9, 10]. Chronic intermittent hypoxia [25, 26], which has been shown to induce liver steatosis [27], is generally considered as a major mechanism through which sleep apnea leads to NAFLD. Nonetheless, we also observed an increased risk of NAFLD in SD patients without sleep apnea. There should be mechanisms other than chronic intermittent hypoxia through which SD may cause NAFLD. Epidemiologic studies have shown that sleep insufficiency may lead to alternation of glucose homeostasis [28], insulin resistance [29, 30], weight gain [31], obesity [32], metabolic syndrome [33, 34], and diabetes mellitus [35, 36], which are all associated with NAFLD [16, 37–39]. In experimental studies, sleep disturbance was found to induce some inflammatory cytokines such as tumor necrosis factor-alpha, interleukin-1 beta, and interleukin-6 [40–42], which play important roles in the pathogenesis of NAFLD [43–45]. Also, sleep loss elevates the level of ghrelin and reduces the level of leptin [46, 47], which increase appetite and further cause obesity. Moreover, chronic insomnia activates the hypothalamo–pituitary–adrenal axis [48], increases stress hormone, worsens insulin resistance, and facilitates the progression of NAFLD [49].

We performed stratified analyses according to most of the generally recognized risk factors for NAFLD, including diabetes mellitus, hypertension, dyslipidemia, depression, and anxiety [19, 50, 51]. The results showed that the effects of SD on the development of NAFLD were significant in patients without these factors, but not in those with these factors. This provided insights that SD may lead to NAFLD through pathways different from those associated with these risk factors and that SD may be a major risk factor for NAFLD in relatively healthy people.

All five of the previous studies on the association between SD and NAFLD that we identified in the literature review adopted cross-sectional study designs. With a longitudinal study design, our study has the advantage of providing stronger evidence of causation in terms of temporal relationship. In addition, most of the previous studies used questionnaires to define SD, and the diagnosis of NAFLD was not confirmed by a physician in all the cases. In the NHANES study [20], for example, the diagnosis of SD was established by questionnaires, and NAFLD was defined as elevated liver enzymes without chronic hepatitis B, chronic hepatitis C, or alcoholic liver disease. In our study, all the diagnoses of SD and NAFLD were made by physicians and subject to routine audits by specialists hired by the National Health Insurance. Furthermore, our study also has the advantage of controlling most of the major potential confounders, including sex, age, and comorbidities of diabetes, hypertension, dyslipidemia, IHD, depression, and anxiety at the same time, which had not been achieved in previous studies. As a result, in addition to SD, we identified male sex, age between 40 and 59 years old, and dyslipidemia as independent risk factors for NAFLD. Because SD and NAFLD are exceedingly common in the general population, we suggest screening programs for NAFLD in patients of SD with above factors.

Our study also has some limitations. First, our study included SD patients who chose to seek medical aid, and therefore the results may not be generalized to the patients who do not seek medical aid. A common reason why SD patients do not seek medical aid is that the illness is not severe. However, studying only the patients who were diagnosed by physicians ensured the accuracy of diagnoses, which is regarded as a strength of our study. Second, although obesity is a well-established risk factor for NAFLD, we were unable to evaluate its effects because the number of patients coded with ICD-9-CM 278 in the NHIRD2000 was small. Again, this was due to the fact that not all patients of obesity would seek medical aids. Nonetheless, we included diabetes, hypertension, and IHD in the analyses, and these conditions are major outcomes of obesity. In other words, the effects of obesity were adjusted indirectly to a certain degree in our analyses. Third, NHIRD2000 does not contain information on sleep pattern, duration, and quality. Therefore, we were unable to study the effects of SD in greater details.

## Conclusions

In this nationwide population-based cohort study, patients with SD had a higher risk of developing NAFLD, including those SD patients who did not have sleep apnea. The association was observed in the subgroups without comorbidities of diabetes, dyslipidemia, hypertension, IHD, depression, or anxiety, but was not observed in the patients who had these comorbidities. This finding indicates that SD may lead to NAFLD through pathways that do not involve these previously recognized risk factors. Further studies are warranted to explore these pathways.

## Data Availability

The data is from the National Health Insurance Research Database (NHIRD), which has been transferred to the Health and Welfare Data Science Center (HWDC). Researchers can apply for the data via HWDC, Department of Statistics, Ministry of Health and Welfare, Taiwan (http://dep.mohw.gov.tw/DOS/np-2497-113.html).

## Abbreviations

AHR: Adjusted hazard ratio
HR: Hazard ratio
ICD-9-CM: International Classification of Diseases, Ninth Revision, Clinical Modification
IHD: Ischemic heart disease
IR: Incidence rate
NAFLD: Nonalcoholic fatty liver disease
NHANES: National Health and Nutrition Examination Survey
NHIRD: National Health Insurance Research Database
SD: Sleep disorders
